# Nonunion of a Stress Fracture at the Base of the Second Metatarsal in a Soccer Player Treated by Osteosynthesis with the Bridging Plate Fixation Technique

**DOI:** 10.1155/2020/6649443

**Published:** 2020-12-22

**Authors:** Futoshi Morio, Shota Morimoto, Shintaro Onishi, Toshiya Tachibana, Tomoya Iseki

**Affiliations:** Department of Orthopedic Surgery, Hyogo College of Medicine, 663-8501, 1-1 Mukogawa-cho, Nishinomiya City, Hyogo, Japan

## Abstract

**Background:**

A stress fracture of the second metatarsal base in soccer players is extremely rare. In this case study, we report a nonunion of a stress fracture at the base of the second metatarsal in a female soccer player who had persistent pain despite continued conservative treatment, who then was treated with the bridging plate fixation technique. *Case Report*. A 19-year-old female college soccer player complained of pain on the dorsum of her right midfoot during a game without history of trauma and was conservatively treated for 6 months. Radiographic examination showed an oblique fracture with small bone fragment at the proximal base of the second metatarsal and computed tomography demonstrated sclerotic change around the fracture site. We diagnosed her with nonunion of a stress fracture at the base of the second metatarsal and performed operative treatments using autogenous cancellous iliac bone grafting and plate fixation bridging a second metatarsal and medial cuneiform with a locking plate. At 4 months after the initial surgery, she was able to return to playing soccer at preinjury level without complications or pain.

**Conclusion:**

We report a rare case of nonunion of a stress fracture at the base of the second metatarsal in a female soccer player without underlying diseases. Surgical treatment was applied, because the conservative treatment was ineffective for 6 months and led to nonunion. The plate fixation technique bridging the second metatarsal and medial cuneiform was a useful option to attain the bone fusion for small fracture fragment for a treatment of nonunion of a stress fracture at the base of the second metatarsal.

## 1. Introduction

Stress fractures occur in around 20% of elite level athletes [1–5], most commonly in the lower limb [6]. Metatarsal stress fractures account for 38% of all stress fractures of the lower limb, and especially, the second and third metatarsal are the most common [7], accounting for 80-90% of all metatarsal fractures [4]. In terms of stress fracture sites, the second metatarsal, the shaft and neck are the most common locations [8]. In regard to the type of sports, stress fractures of the second metatarsal most commonly occur in classical ballet dancers [9].

As for treatment for stress fractures at the base of the second metatarsal base, conservative treatment such as immobilization, restricted weight bearing, and physical therapy is generally recommended as initial treatment [10], and good results have been reported [11–14]. In cases of persistent pain and delayed or nonunion despite these treatments, an operative treatment such as plate fixation with bone grafting is considered as a treatment option [9, 15, 16]. However, it is still unclear which surgical treatment is the optimal choice.

We report a rare case of nonunion of a stress fracture at the base of the second metatarsal in a female soccer player who had persistent pain despite continued conservative treatment, who then was treated with the bridging plate fixation technique. She was able to return to playing soccer at preinjury level at 4 months after surgery, and no recurring symptoms nor refracture were present at the 2 years follow-up. Written informed consent was obtained from the patient for publishing this report. For author contribution, SM, FM, and SO performed surgery. They were involved in data acquisition and followed patients in clinics. TI and TT helped to draft the manuscript and participated in the design of the study. All authors read and approved the final manuscript.

## 2. The Case

**Figure 1:**
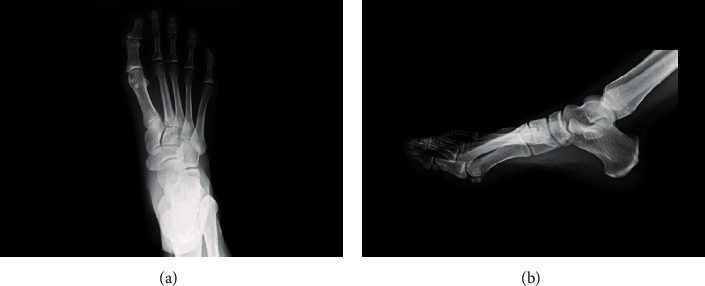
Preoperative foot radiograph at an initial visit to our institution. An oblique fracture line at the base of the second metatarsal was identified on (a) anteroposterior and (b) lateral views.

**Figure 2:**
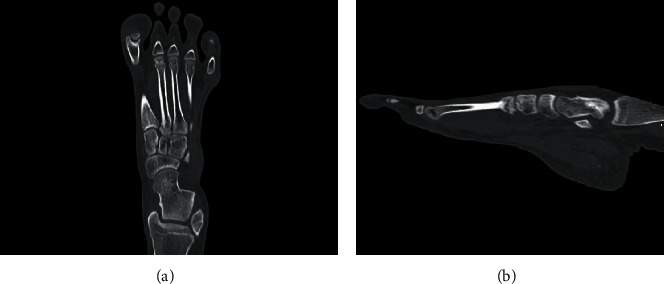
Preoperative CT demonstrated a fracture line at the base of the second metatarsal and sclerotic change around the fracture site on (a) coronal and (b) sagittal views.

A 19-year-old female college soccer player complained of pain on the dorsum of her right midfoot during a game without history of trauma. Her height, weight, and body mass index were 158 cm, 58 kg, and 23.2 kg/m^2^, respectively. She did not have any medical history or menstrual abnormalities. Furthermore, she trained 5 times a week for 3 hours each and played as a defender in games once a week. After her midfoot pain developed, she visited a local clinic and was diagnosed with stress fracture at the base of the second metatarsal. Although she was treated with conservative treatment including rest, restricted weight bearing, and physical therapy for 6 months, her midfoot pain remained, and radiological findings demonstrated no evidence of bone union at the fracture site, which led her to visiting our institution. At the first visit to our institution, she could not play soccer due to her midfoot pain, and physical examination revealed tenderness at the base of the second metatarsal. The American Orthopedic Foot and Ankle Society Midfoot Scale (AOFAS) score (100 points) was 70 points at that time. Radiographic examination showed an oblique fracture line at the base of the second metatarsal (Figure 1). Magnetic resonance imaging (MRI) showed a linear hypointense fracture line on T1-weighted and T2-weighted images and a bone marrow edema around the fracture site on STIR. Computed tomography (CT) demonstrated sclerotic change around the fracture site (Figure 2). In addition, laboratory examination revealed no abnormal findings such as iron deficiency anemia and vitamin D deficiency. According to clinical history, physical examination, and radiological findings, we diagnosed her with nonunion of a stress fracture at the base of the second metatarsal. Owing to the fact that the conservative treatment was ineffective for 6 months, we recommended operative treatment.

## 3. Surgical Technique

**Figure 3:**
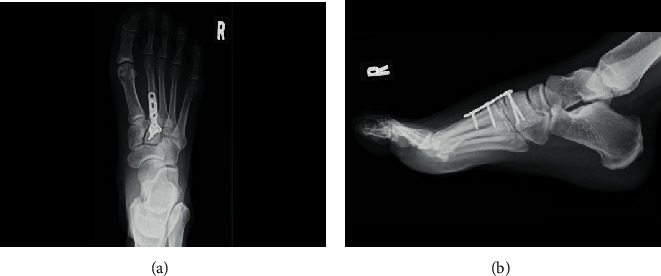
Postoperative (a) anteroposterior and (b) lateral foot radiographs. The nonunion site was fixed using the bridging plate fixation technique with a locking plate, which was placed between the distal fragment of the second metatarsal and the medial cuneiform.

The operation was performed under general anesthesia in the supine position. An air tourniquet and a fluoroscopy were used for the operation. An approximately 4 cm straight incision was placed at the dorsal side of the tarsometatarsal joint. After confirming the nonunion lesion at the second metatarsal, the bone lesion was removed. An autogenous cancellous bone was harvested from the ipsilateral iliac bone using a minimally invasive technique with the Autograft OATS System (Arthrex, NJ, USA), then inserted into the union site, and subsequently fixed using the bridging plate fixation technique with a locking plate (T-fusion Plate, DePuySynthes, NJ, USA), which was placed between the distal fragment of the second metatarsal and the medial cuneiform **(**Figure 3). The wound was sutured, and the operation was concluded.

**Figure 4:**
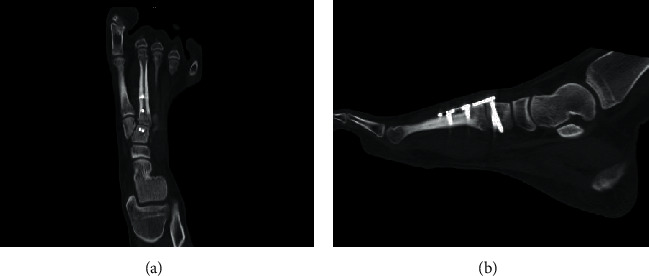
At 12 weeks after the operation, CT demonstrated complete bone union at the fracture site on (a) coronal and (b) sagittal views.

Postoperatively, a nonweightbearing short leg splint was applied for 2 weeks, and when the splint was removed, active range of motion exercises of the ankle was initiated. Partial weightbearing was allowed at 4 weeks postoperatively and full weightbearing at 8 weeks postoperatively. At 4 months after the initial surgery, she was able to return to playing soccer at preinjury level without pain. She had no recurring symptoms and no refracture at 2 years postoperatively. AOFAS score improved to 100 points, and the patient was completely satisfied with the result. The locking plate was removed after confirming complete bone union on CT at 12 weeks after the operation (Figure 4).

## 4. Discussion

In this report, we have described a rare case of nonunion of a stress fracture at the base of the second metatarsal in a female soccer player who had persistent pain despite continued conservative treatment, then received operation. This athlete was able to return to playing soccer at preinjury level at 4 months after the operation using the bridging plate fixation technique, and no recurring symptoms nor refracture were present at the 2 years follow-up.

Stress fractures of the second metatarsal are common injuries due to overuse in athletes, and most of them occur at the shaft and the neck of the second metatarsal [8]. On the other hand, stress fractures at the base of the second metatarsal are relatively rare and account for less than 5% of all second metatarsal stress fractures [17]. It has been reported that most of the stress fractures at the second metatarsal base occur in classical ballet dancers. Previous reports demonstrated that anatomical factors and characteristics of classical ballet are involved in this fact. Anatomically, the second metatarsal base is surrounded by the first, second, and third cuneiform, constituting the mortise joint [17]. In addition, the extreme plantar flexion of the foot and ankle in the “en pointe” position is repeated in classical ballet dance. Due to the anatomical features and the characteristics of the competition, the base of the second metatarsal in classical ballet dancers easily undergoes repetitive stress. On the other hand, stress fractures of the second metatarsal base are extremely rare in nondancers. To our knowledge, there is only one report of stress fractures in nondancers, which involved 9 cases, all with underlying diseases with risk of stress fractures such as chronic steroid usage, calcium and phosphate metabolic disease, and female athlete triple triad [9]. However, there are no reports of stress fractures at the base of the second metatarsal in nondancers without underlying diseases.

In addition, several reports showed that cases with patients who have a short first metatarsal compared to the second metatarsal increases the risk of a second metatarsal stress fracture [11, 18]. These reports showed that the average length of the first metatarsal in patients with second proximal metatarsal stress fractures was approximately 80 to 82% of the second metatarsal [8, 11, 18].

There are several reports on treatments for stress fractures at the base of the second metatarsal. The optimal treatment is still under debate; however, conservative treatment including immobilization, restricted weight-bearing, and physical therapy is generally recommended as the initial treatment. Although a success rate of 75 to 100% have been reported for conservative treatment in previous literatures [11–14], treatment failures such as delayed union and nonunion can potentially occur [15]. Surgical treatment is generally indicated for these treatment failures [19]. There are only three previous reports on surgical treatment for nonunion of stress fractures at the base of the second metatarsal [9, 15, 16]. Plate fixation with bone grafting is one of the most common surgical methods with good results. Muscolo et al. [15] performed osteosynthesis between the proximal and distal fragment using a locking plate for a male ballet dancer who progressively returned to full-time dancing without symptoms at 3 months after surgery. Chuckpaiwong et al. [9] also performed plate fixation with allogeneic bone grafting for a female nondancer with diabetes and hypophosphatasia, and good union was obtained. In general, surgical outcomes with plate fixation generally achieve good results; however, in handling with small proximal fragment of second metatarsal, it might be difficult to fix both the distal and proximal fragments using a plate. To attain the bone fusion for small fracture fragment, the effectiveness of the bridging plate fixation technique has been reported [20, 21]. To our knowledge, there are no reports on surgical treatment using the bridging plate fixation technique for nonunion of stress fractures at the base of the second metatarsal.

The patient in this report was a soccer player in the defender position which indicated that she had a higher frequency of jumping than other players. For this reason, the base of the second metatarsal might have undertaken repetitive stress. In addition, the length of the first metatarsal was 79% of that of the second metatarsal, which is comparatively shorter than usual. Therefore, the morphologic abnormality of the first metatarsal was raised as a risk factor.

Operative treatment using the bridging plate fixation technique was performed since the proximal fragment was small. We suggest this technique to fix the nonunion site more easily in cases that the proximal fragment is small due to stress fractures at the base of the second metatarsal.

## 5. Conclusion

We reported a case of nonunion of a stress fracture at the base of the second metatarsal in a female soccer player without underlying diseases. In this case, we speculated that the stress fracture was caused by a playing style that involves a high frequency of jumping and a morphologic abnormality of a short first metatarsal. Furthermore, surgical treatment was indicated in this case, because the conservative treatment was ineffective for 6 months and led to nonunion. Osteosynthesis with the bridging plate fixation technique was a useful option for nonunion of a stress fracture at the base of the second metatarsal.
